# Supplementary material for: Identification of genes critical for inducing ulcerative colitis and exploring their tumorigenic potential in human colorectal carcinoma
Source: PLoS One. 2023 Aug 3;18(8):e0289064. doi: 10.1371/journal.pone.0289064 (PMC10399749; doi:10.1371/journal.pone.0289064)
Supplement: S1 File — (DOCX) [file pone.0289064.s001.docx]

**Supporting Information – contains all the supporting tables and figures**


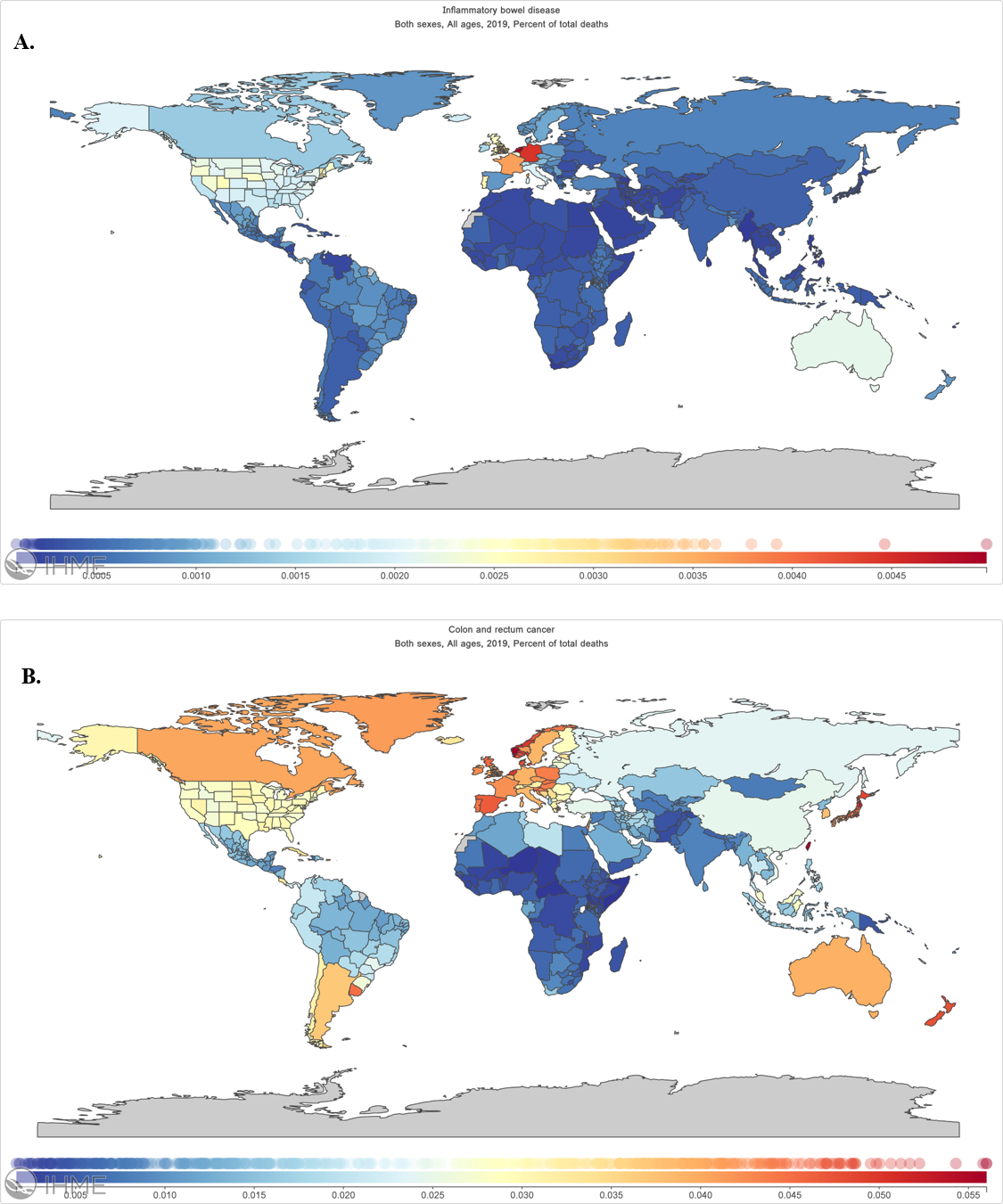
Supplementary Figures

Fig.S1. The Global Burden of Disease (GBD) study of 2019 for IBD and cancer associated with colon and rectum


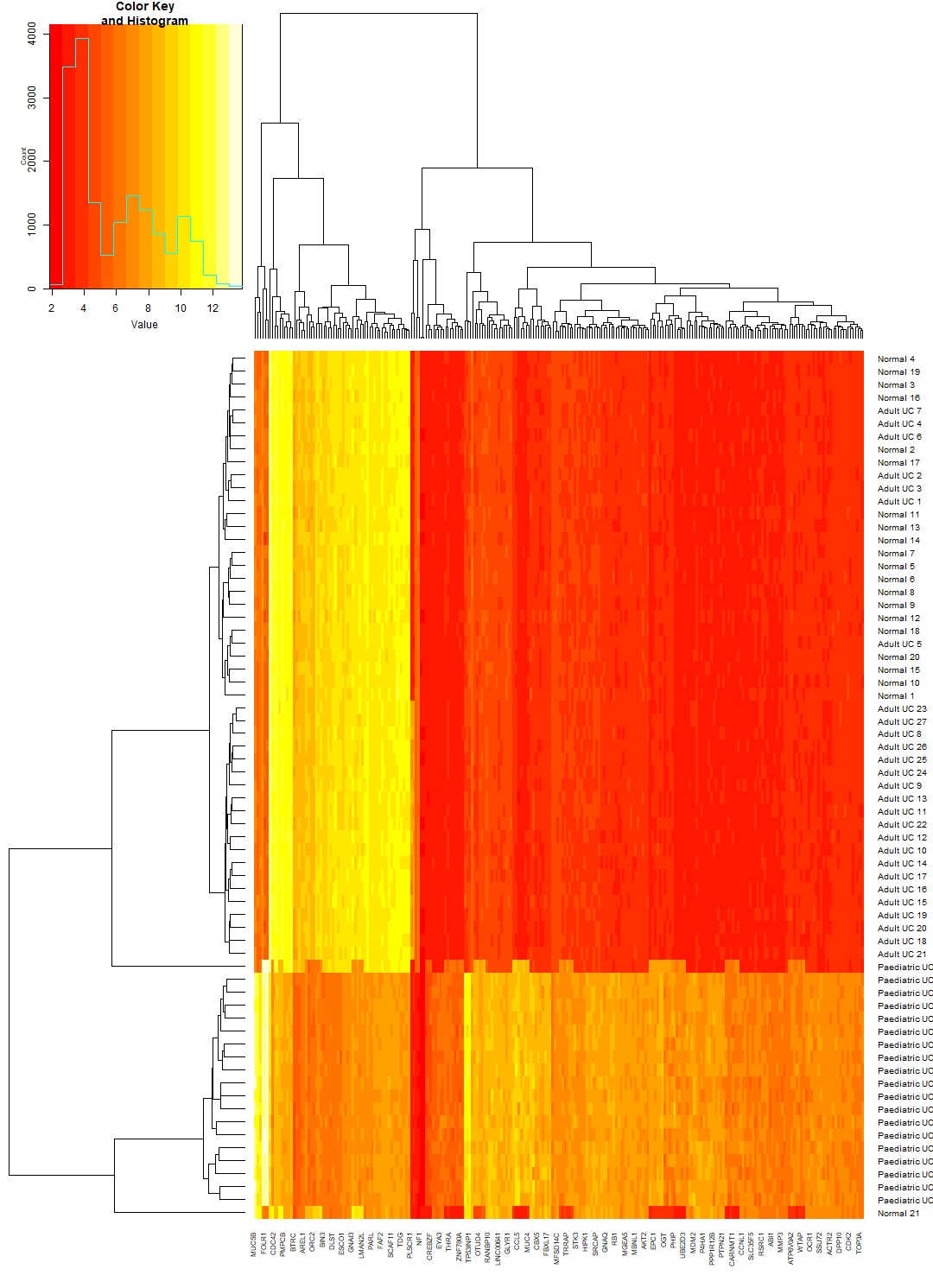


Fig.S2. Clustered heatmap showing the expression patterns of top 250 differentially expressed genes in normal/healthy control, paediatric UC and adult UC samples.


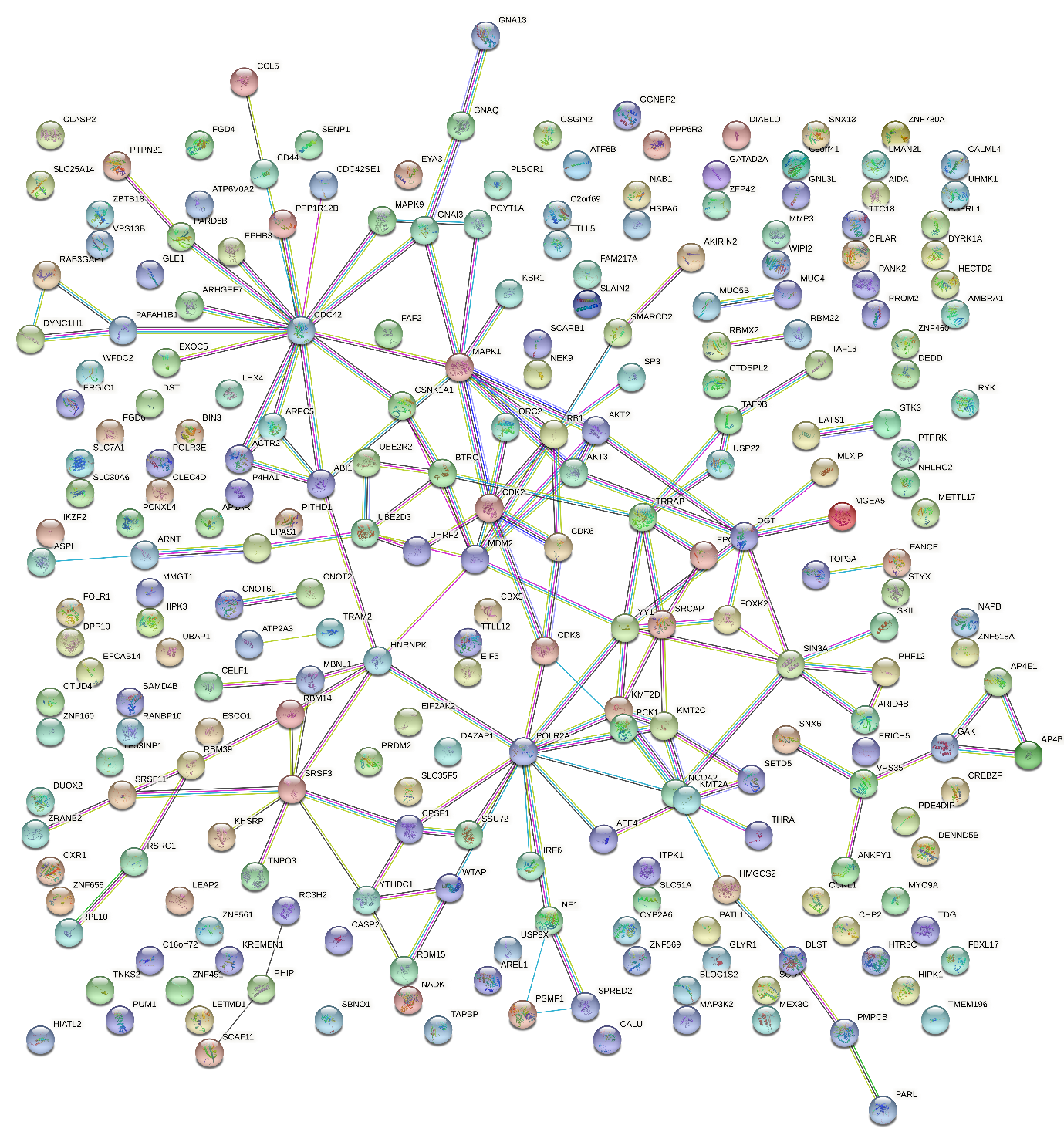


Fig.S3. Protein-protein interactions (PPI) network of the gene products of top 250 DEGs obtained from the three different groups of subjects viz, normal, pediatric UC, and adult UC was analysed using STRING.


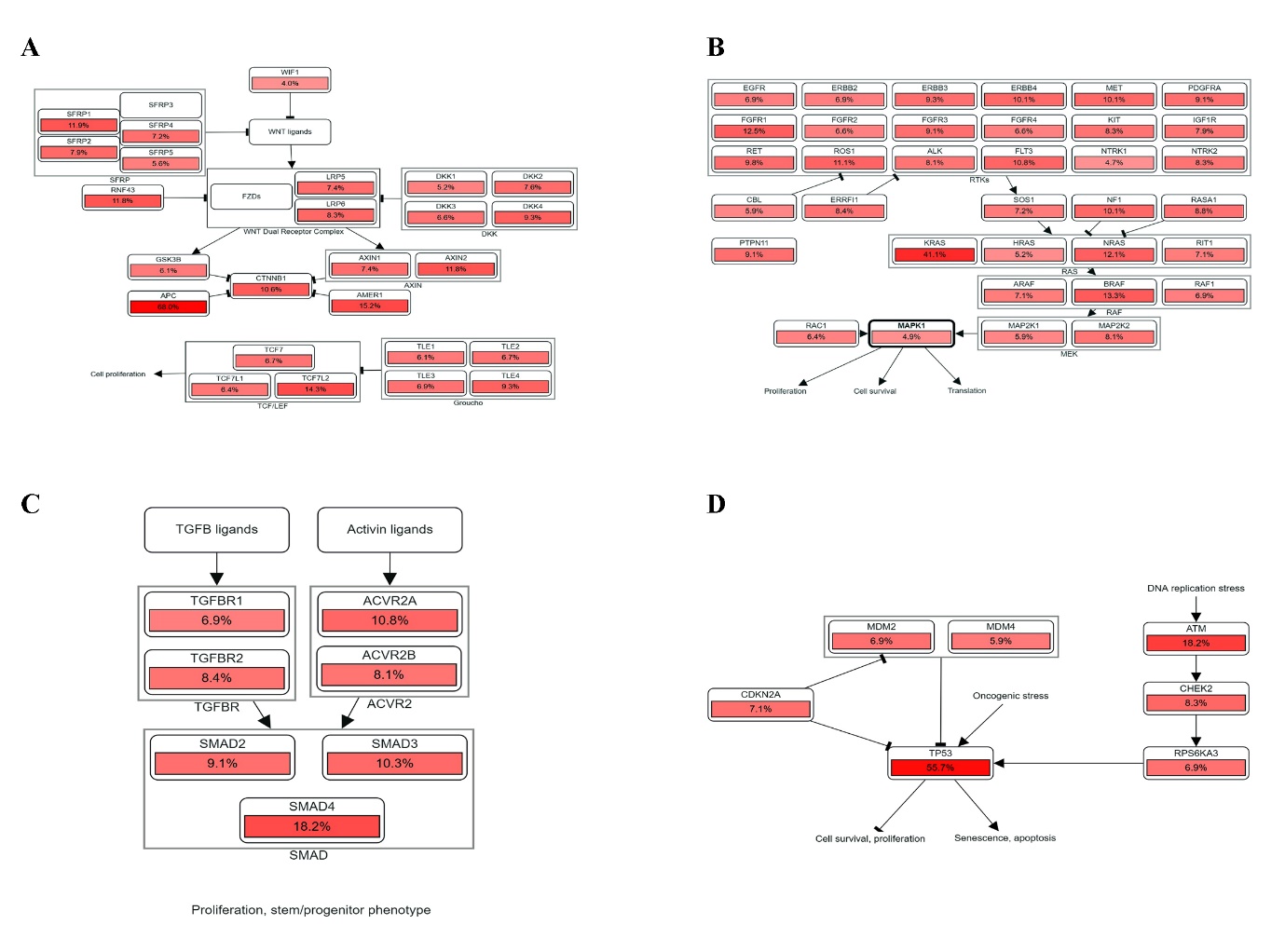


Fig.S4. Determining the relative influence of molecular alterations in UC-critical genes over the various signalling pathways associated with CRC. Frequency of alterations in the different signalling components of A. Wnt signalling pathway, B. RTK-RAS pathways, C. TGF-β signalling pathway, and D. TP53 signalling pathway impacted by the UC-critical genes was explored using Pathway mapper of cBioPortal.


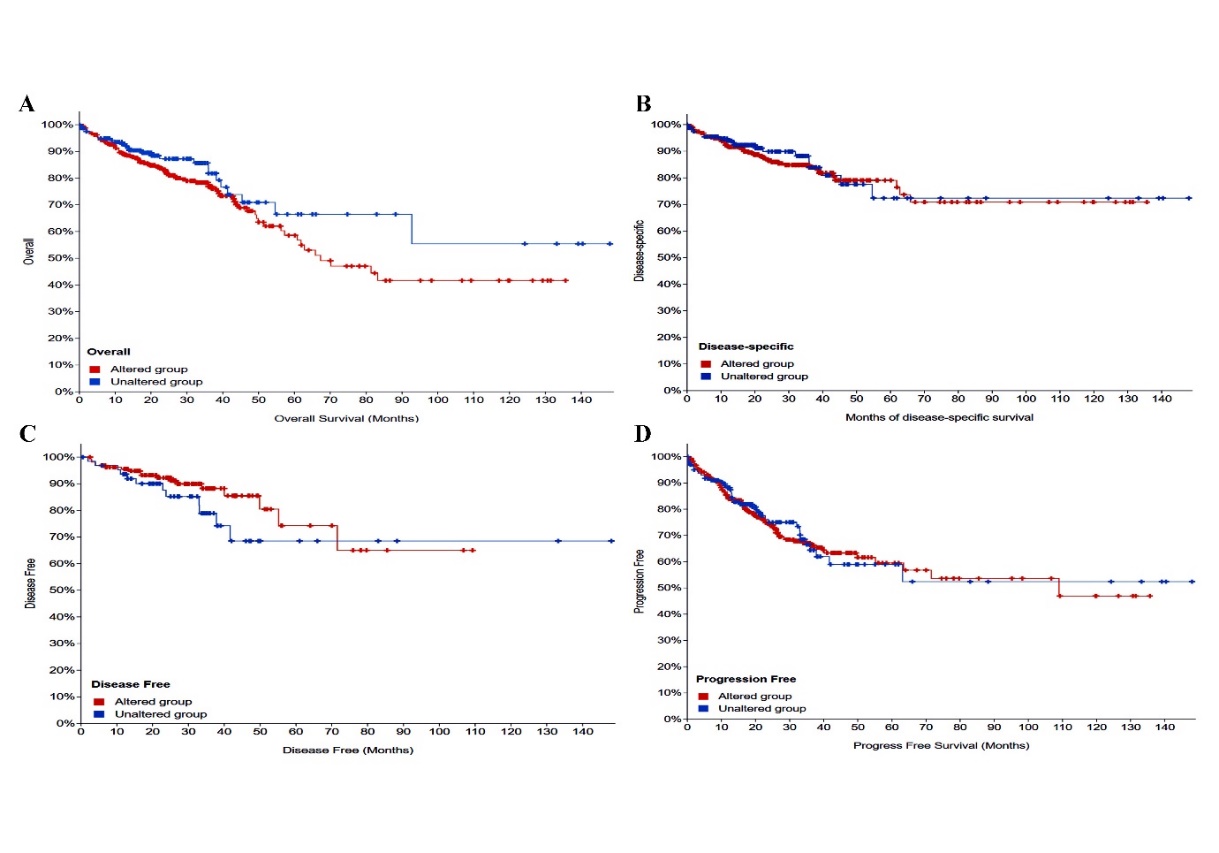


Fig.S5. Determining the impact of altered 24 UC-critical genes over the survival of patients with CRC. KM plots of altered and unaltered 24 UC-critical genes depicting the A. Overall survival, B. Disease-specific survival, C. Disease-free survival, D. Progression-free survival in CRC. Red and blue colour respectively indicates the groups having altered and unaltered expressions of the UC-critical genes.

SUPPLEMENTARY TABLES

Table.S1. Clinical characteristics of the participants.

| Characteristics | Adult Ulcerative Colitis Patient | Pediatric Ulcerative Colitis Patient |
| --- | --- | --- |
| Age (in years) |  |  |
| Range | 19-69 | 6-17 |
| Median | 32 | 15 |
| Interquartile range  Q1  Q2 | 27  50 | 10  16 |
| Extent of Disease |  |  |
| Limited (n) | 0 | 6 |
| Extensive (n) | 27 | 13 |

Table.S2. List of top 250 differentially expressed genes (DEGs) across the three different groups viz., normal tissue, paediatric ulcerative colitis tissue, and adult ulcerative colitis tissue.

| SL. No. | Gene List | *Status of Gene expression in UC patient population | |
| --- | --- | --- | --- |
|  |  | Paediatric population (6-17 years) | Adult population (18-69 years) |
| 1. | MAPK9 | UP | DOWN |
| 2 | ZNF460 | UP | DOWN |
| 3 | MUC4 | UP | DOWN |
| 4 | PTPRK | UP | DOWN |
| 5 | PITHD1 | UP | DOWN |
| 6 | PDE4DIP | UP | DOWN |
| 7 | PATL1 | UP | DOWN |
| 8 | MAP3K2 | UP | DOWN |
| 9 | MAPK1 | UP | UP |
| 10 | CCL5 | UP | UP |
| 11 | DYRK1A | UP | UP |
| 12 | SCARB1 | DOWN | UP |
| 13 | VPS35 | UP | UP |
| 14 | TTLL12 | DOWN | UP |
| 15 | UBE2D3 | UP | UP |
| 16 | RBM39 | UP | UP |
| 17 | HMGCS2 | DOWN | UP |
| 18 | SLC51A | DOWN | UP |
| 19 | ERGIC1 | UP | UP |
| 20 | THRA | UP | UP |
| 21 | CNOT6L | UP | UP |
| 22 | PLSCR1 | UP | DOWN |
| 23 | ZNF451 | UP | DOWN |
| 24 | WFDC2 | DOWN | UP |
| 25 | EPHB3 | UP | UP |
| 26 | MDM2 | UP | DOWN |
| 27 | RPL10 | DOWN | UP |
| 28 | PTPN21 | UP | DOWN |
| 29 | AKT2 | UP | DOWN |
| 30 | SMARCD2 | DOWN | UP |
| 31 | PCK1 | UP | DOWN |
| 32 | SLC35F5 | UP | DOWN |
| 33 | RB1 | UP | DOWN |
| 34 | SBNO1 | UP | DOWN |
| 35 | CBX5 | UP | DOWN |
| 36 | NCOA2 | UP | DOWN |
| 37 | C16orf72 | UP | DOWN |
| 38 | DPP10 | UP | DOWN |
| 39 | FBXL17 | UP | DOWN |
| 40 | GNAQ | UP | DOWN |
| 41 | LOC100507412 | UP | DOWN |
| 42 | FGD6 | UP | UP |
| 43 | MUC5B | UP | DOWN |
| 44 | KMT2D | UP | DOWN |
| 45 | GNA13 | UP | UP |
| 46 | CLASP2 | UP | DOWN |
| 47 | TNPO3 | UP | DOWN |
| 48 | PAFAH1B1 | DOWN | DOWN |
| 49 | CTDSPL2 | UP | DOWN |
| 50 | LINC00641 | UP | DOWN |
| 51 | GAK | UP | DOWN |
| 52 | GATAD2A | UP | UP |
| 53 | MLXIP | UP | DOWN |
| 54 | P4HA1 | UP | DOWN |
| 55 | PPP1R12B | UP | UP |
| 56 | NADK | DOWN | UP |
| 57 | PUM1 | UP | DOWN |
| 58 | TDG | DOWN | DOWN |
| 59 | KMT2C | UP | DOWN |
| 60 | USP22 | UP | DOWN |
| 61 | POLR2A | UP | DOWN |
| 62 | FAM217A | UP | DOWN |
| 63 | STYX | UP | DOWN |
| 64 | LOC283745 | UP | DOWN |
| 65 | RBM14 | DOWN | UP |
| 66 | FANCE | DOWN | UP |
| 67 | FGFRL1 | UP | DOWN |
| 68 | ACTR2 | UP | DOWN |
| 69 | ZNF655 | UP | UP |
| 70 | ARNT | UP | DOWN |
| 71 | AIDA | UP | DOWN |
| 72 | TP53INP1 | UP | DOWN |
| 73 | PANK2 | DOWN | UP |
| 74 | CFLAR | UP | DOWN |
| 75 | CASP2 | UP | DOWN |
| 76 | SRSF3 | UP | DOWN |
| 77 | AKIRIN2 | UP | DOWN |
| 78 | UHMK1 | UP | DOWN |
| 79 | ZNF252P | DOWN | DOWN |
| 80 | GLYR1 | UP | DOWN |
| 81 | PMPCB | DOWN | DOWN |
| 82 | GGNBP2 | UP | DOWN |
| 83 | SSU72 | UP | DOWN |
| 84 | RYK | DOWN | UP |
| 85 | SPRED2 | DOWN | UP |
| 86 | AREL1 | DOWN | UP |
| 87 | AKT3 | UP | DOWN |
| 88 | AP1AR | UP | DOWN |
| 89 | SNX6 | DOWN | UP |
| 90 | ZRANB2 | UP | DOWN |
| 91 | C2orf69 | UP | DOWN |
| 92 | MGEA5 | UP | DOWN |
| 93 | PCYT1A | DOWN | UP |
| 94 | AFF4 | UP | DOWN |
| 95 | PARL | DOWN | UP |
| 96 | ERICH5 | DOWN | UP |
| 97 | EIF2AK2 | UP | DOWN |
| 98 | IRF6 | UP | DOWN |
| 99 | LHX4 | UP | DOWN |
| 100 | RSRC1 | UP | DOWN |
| 101 | RBM15 | DOWN | UP |
| 102 | SLC25A14 | DOWN | UP |
| 103 | HIPK1 | UP | DOWN |
| 104 | LATS1 | UP | DOWN |
| 105 | CDK8 | DOWN | UP |
| 106 | EPAS1 | UP | UP |
| 107 | MBNL1 | UP | UP |
| 108 | ZNF460 | UP | DOWN |
| 109 | MEX3C | UP | DOWN |
| 110 | CYP2A6 | UP | DOWN |
| 111 | MMGT1 | DOWN | UP |
| 112 | OSGIN2 | UP | DOWN |
| 113 | DST | UP | DOWN |
| 114 | CNOT2 | DOWN | UP |
| 115 | CDC42 | DOWN | UP |
| 116 | HNRNPK | DOWN | DOWN |
| 117 | EYA3 | UP | DOWN |
| 118 | NHLRC2 | UP | DOWN |
| 119 | ARID4B | UP | DOWN |
| 120 | CDC42SE1 | UP | DOWN |
| 121 | PARD6B | UP | DOWN |
| 122 | DIABLO | DOWN | UP |
| 123 | MFSD14C | UP | DOWN |
| 124 | TAF13 | UP | DOWN |
| 125 | SNX13 | UP | DOWN |
| 126 | SCAF11 | DOWN | UP |
| 127 | HIPK3 | DOWN | UP |
| 128 | DLST | DOWN | UP |
| 129 | CFAP70 | UP | DOWN |
| 130 | USP9X | UP | DOWN |
| 131 | CLEC4D | DOWN | UP |
| 132 | CDK2 | UP | DOWN |
| 133 | ZNF518A | UP | DOWN |
| 134 | TNKS2 | UP | DOWN |
| 135 | MYO9A | UP | DOWN |
| 136 | ANKFY1 | DOWN | UP |
| 137 | SAMD4B | UP | DOWN |
| 138 | TMEM196 | UP | DOWN |
| 139 | ZNF569 | DOWN | UP |
| 140 | CALU | UP | DOWN |
| 141 | UBE2R2 | DOWN | UP |
| 142 | FAF2 | DOWN | UP |
| 143 | RC3H2 | UP | DOWN |
| 144 | PROM2 | UP | DOWN |
| 145 | CCNL1 | UP | DOWN |
| 146 | ATP2A3 | UP | DOWN |
| 147 | GLE1 | DOWN | UP |
| 148 | EIF5 | DOWN | UP |
| 149 | RANBP10 | UP | DOWN |
| 150 | ARPC5 | UP | DOWN |
| 151 | SIN3A | UP | DOWN |
| 152 | PRDM2 | UP | DOWN |
| 153 | TOP3A | UP | DOWN |
| 154 | LEAP2 | UP | UP |
| 155 | OXR1 | UP | DOWN |
| 156 | ZBTB18 | UP | DOWN |
| 157 | ABI1 | UP | DOWN |
| 158 | VPS13B | UP | DOWN |
| 159 | UBAP1 | DOWN | UP |
| 160 | SRCAP | UP | DOWN |
| 161 | ZNF561 | DOWN | UP |
| 162 | WIPI2 | UP | DOWN |
| 163 | IKZF2 | UP | DOWN |
| 164 | DYNC1H1 | UP | DOWN |
| 165 | ESCO1 | DOWN | UP |
| 166 | CPSF1 | UP | DOWN |
| 167 | PCNX4 | UP | DOWN |
| 168 | FGD4 | UP | DOWN |
| 169 | ITPK1 | UP | DOWN |
| 170 | ARHGEF7 | DOWN | UP |
| 171 | RBM22 | DOWN | UP |
| 172 | POLR3E | UP | DOWN |
| 173 | BIN3 | DOWN | UP |
| 174 | OCR1 | UP | DOWN |
| 175 | TAPBP | UP | DOWN |
| 176 | CD44 | UP | DOWN |
| 177 | DEDD | DOWN | UP |
| 178 | SETD5 | DOWN | UP |
| 179 | SCD | UP | DOWN |
| 180 | LETMD1 | UP | DOWN |
| 181 | NEK9 | UP | DOWN |
| 182 | BTRC | DOWN | UP |
| 183 | ZFP42 | UP | DOWN |
| 184 | SRSF11 | UP | DOWN |
| 185 | BLOC1S2 | DOWN | UP |
| 186 | YTHDC1 | DOWN | UP |
| 187 | SLC30A6 | UP | DOWN |
| 188 | MMP3 | UP | UP |
| 189 | CALML4 | UP | DOWN |
| 190 | USP9X | DOWN | UP |
| 191 | DUOX2 | DOWN | UP |
| 192 | KSR1 | UP | DOWN |
| 193 | STK3 | UP | DOWN |
| 194 | SENP1 | UP | DOWN |
| 195 | DAZAP1 | DOWN | UP |
| 196 | WTAP | UP | DOWN |
| 197 | ORC2 | DOWN | UP |
| 198 | EXOC5 | UP | DOWN |
| 199 | CDK6 | UP | DOWN |
| 200 | HECTD2 | UP | DOWN |
| 201 | LOC403312 | UP | DOWN |
| 202 | PHF12 | UP | DOWN |
| 203 | SLAIN2 | UP | DOWN |
| 204 | HTR3C | UP | DOWN |
| 205 | TRRAP | UP | DOWN |
| 206 | KMT2A | UP | DOWN |
| 207 | NAB1 | UP | DOWN |
| 208 | CREBZF | UP | DOWN |
| 209 | YY1 | DOWN | UP |
| 210 | KHSRP | UP | DOWN |
| 211 | ZNF160 | UP | DOWN |
| 212 | OTUD4 | UP | DOWN |
| 213 | PSMF1 | DOWN | UP |
| 214 | GNAI3 | DOWN | UP |
| 215 | METTL17 | DOWN | UP |
| 216 | CELF1 | UP | UP |
| 217 | AMBRA1 | UP | UP |
| 218 | SKIL | UP | UP |
| 219 | ATP6V0A2 | UP | DOWN |
| 220 | LMAN2L | DOWN | UP |
| 221 | ASPH | UP | UP |
| 222 | FOLR1 | UP | DOWN |
| 223 | HSPA6 | UP | UP |
| 224 | SP3 | UP | DOWN |
| 225 | NF1 | DOWN | UP |
| 226 | PPP6R3 | DOWN | UP |
| 227 | KREMEN1 | UP | DOWN |
| 228 | RBMX2 | DOWN | UP |
| 229 | AP4B1 | UP | UP |
| 230 | AP4E1 | DOWN | UP |
| 231 | CSNK1A1 | UP | UP |
| 232 | RAB3GAP1 | UP | UP |
| 233 | NUS1P3 | UP | UP |
| 234 | TAF9B | UP | DOWN |
| 235 | GNL3L | UP | DOWN |
| 236 | SLC7A1 | UP | UP |
| 237 | CARNMT1 | UP | DOWN |
| 238 | UHRF2 | DOWN | UP |
| 239 | DENND5B | UP | DOWN |
| 240 | TRAM2 | UP | UP |
| 241 | ATF6B | DOWN | UP |
| 242 | CHP2 | DOWN | UP |
| 243 | TTLL5 | DOWN | UP |
| 244 | ZNF780A | UP | DOWN |
| 245 | FOXK2 | UP | DOWN |
| 246 | EPC1 | UP | DOWN |
| 247 | OGT | UP | DOWN |
| 248 | EFCAB14 | UP | DOWN |
| 249 | PHIP | UP | UP |
| 250 | NAPB | DOWN | UP |

*Expression profiles were determined from the transcriptome of the UC patient population with respect to the healthy control.

Table.S3. Gene Ontology and functional enrichment analysis of top 250 DEGs involved in the induction of UC using DAVID server.

| Terms | % | Associated Genes | P-value | Fold Enrichment |
| --- | --- | --- | --- | --- |
| GO: BIOLOGICAL PROCESSES | | | | |
| GO:0045944~positive regulation of transcription from RNA polymerase II promoter | 14.2 | RB1, CREBZF, KMT2D, ZNF451, SMARCD2, KMT2A, THRA, ATF6B, EPAS1, KMT2C, FOXK2, ARID4B, PRDM2, YY1, SIN3A, CHP2, EPC1, MLXIP, ZBTB18, NCOA2, AKIRIN2, ACTR2, RBM14, ARNT, SENP1, CDK8, PLSCR1, HNRNPK, SP3, MDM2, PHIP, IRF6, LHX4, OGT, GLYR1 | 2.78E-06 | 2.415760598 |
| GO:0006357~regulation of transcription from RNA polymerase II promoter | 14.2 | RB1, CREBZF, SMARCD2, THRA, SRCAP, ATF6B, TRRAP, EPAS1, FOXK2, ZNF518A, ARID4B, PRDM2, IKZF2, YY1, ZNF569, EPC1, CCNL1, MLXIP, ZBTB18, ZNF561, ZNF460, USP22, ZNF160, ARNT, HNRNPK, CNOT2, SP3, ZFP42, MDM2, PHIP, IRF6, LHX4, ZNF780A, ZNF655, OGT | 0.002607 | 1.689481142 |
| GO:0000122~negative regulation of transcription from RNA polymerase II promoter | 10.1 | RB1, THRA, RPL10, UBE2D3, FOXK2, PRDM2, YY1, SIN3A, TDG, ORC2, EPC1, TAF9B, SKIL, ZBTB18, NCOA2, AKIRIN2, CBX5, USP9X, PHF12, GATAD2A, CDK6, CNOT2, SP3, CDK2, MDM2 | 5.72E-04 | 2.157829702 |
| GO:0006355~regulation of transcription, DNA-templated | 8.9 | RB1, NCOA2, ZNF561, KMT2D, ZNF460, TRRAP, ZNF160, KMT2C, PHF12, NAB1, FOXK2, PRDM2, ARID4B, SBNO1, POLR2A, CNOT2, KHSRP, TP53INP1, SP3, ZNF569, ZNF780A, ZNF655 | 0.007157 | 1.869616395 |
| GO:0007165~signal transduction | 8.9 | CDC42SE1, KSR1, ATF6B, CSNK1A1, EPAS1, RYK, ITPK1, HTR3C, PTPRK, OSGIN2, STK3, GNA13, HNRNPK, AKT2, AKT3, CDK2, NF1, MAPK1, ARHGEF7, BTRC, PPP1R12B, OGT | 0.056519 | 1.510910924 |
| GO:0006468~protein phosphorylation | 8.5 | MAP3K2, NEK9, KSR1, TAF13, CSNK1A1, DYRK1A, EIF2AK2, HIPK1, HIPK3, STK3, CDC42, GAK, LATS1, CDK8, MAPK9, CDK6, RSRC1, CCL5, AKT3, CDK2, MAPK1 | 3.43E-06 | 3.438512313 |
| GO:0045893~positive regulation of transcription, DNA-templated | 8.1 | MAP3K2, SMARCD2, KMT2A, SRCAP, USP22, DYRK1A, ARNT, FOXK2, YY1, ASPH, PITHD1, BLOC1S2, TP53INP1, SP3, CDK2, MAPK1, EPC1, PHIP, IRF6, BTRC | 4.79E-04 | 2.479401325 |
| GO:0045892~negative regulation of transcription, DNA-templated | 6.9 | RB1, ZBTB18, CREBZF, CBX5, THRA, PHF12, NAB1, FOXK2, PTPRK, GATAD2A, HNRNPK, SIN3A, SP3, MDM2, EPC1, BTRC, SNX6 | 0.001849 | 2.423614795 |
| GO:0051726~regulation of cell cycle | 6.0 | RB1, UHRF2, TRRAP, FBXL17, PUM1, UHMK1, YY1, CDK8, CDK6, AKT2, TP53INP1, MDM2, EPC1, BTRC, SKIL | 3.01E-06 | 4.826149855 |
| GO:0006915~apoptotic process | 6.0 | AREL1, KMT2A, DIABLO, UBE2D3, KREMEN1, CFLAR, HIPK3, STK3, PLSCR1, TP53INP1, MDM2, CASP2, MAPK1, OGT, DEDD | 0.011354 | 2.123836495 |
| GO:0043066~negative regulation of apoptotic process | 5.6 | AREL1, RPL10, EIF2AK2, CFLAR, HIPK3, HNRNPK, SIN3A, AKT2, MDM2, CASP2, PHIP, LHX4, TAF9B, CD44 | 0.012765 | 2.171918346 |
| GO:0051301~cell division | 5.2 | RB1, NEK9, DYNC1H1, CSNK1A1, USP9X, GNAI3, CDC42, LATS1, PARD6B, CDK6, CDK2, CLASP2, PAFAH1B1 | 0.002339 | 2.821376495 |
| GO:0008285~negative regulation of cell proliferation | 5.2 | GGNBP2, CELF1, EIF2AK2, PTPRK, AMBRA1, FGFRL1, STK3, CDK6, ASPH, TP53INP1, ABI1, NF1, IRF6 | 0.009694 | 2.352176027 |
| GO:0016032~viral process | 5.2 | RB1, CBX5, RBM15, SRCAP, DYRK1A, CFLAR, POLR2A, ABI1, MDM2, MAPK1, VPS35, BTRC, OGT | 0.012713 | 2.267815284 |
| GO:0018105~peptidyl-serine phosphorylation | 4.8 | LATS1, MAPK9, CSNK1A1, AKT2, AKT3, CDK2, DYRK1A, MAPK1, HIPK1, PCK1, HIPK3, UHMK1 | 8.30E-06 | 5.735586186 |
| GO: MOLECULAR FUNCTIONS | | | | |
| GO:0005515~protein binding | 79.2 | RB1, TRRAP, IKZF2, GLE1, AKT2, AKT3, ANKFY1, EPC1, VPS35, CCNL1, BTRC, EPHB3, MBNL1, SCAF11, SLC51A, UHMK1, BIN3, NHLRC2, TTLL12, LETMD1, SRCAP, LMAN2L, EPAS1, SLAIN2, PCNX4, BLOC1S2, ATP6V0A2, PSMF1, SNX6, PROM2, ZRANB2, EIF2AK2, PUM1, MEX3C, HNRNPK, EIF5, RANBP10, CALU, DAZAP1, FAF2, AP4E1, PTPRK, TRAM2, WFDC2, C16ORF72, RSRC1, NCOA2, PCYT1A, CNOT6L, TOP3A, KREMEN1, GAK, PLSCR1, ZNF780A, AIDA, EXOC5, ARHGEF7, ERGIC1, CD44, PAFAH1B1, NAPB, RPL10, GNAI3, DLST, SLC7A1, TAF9B, SRSF11, DUOX2, DEDD, MAP3K2, DYNC1H1, ZNF460, USP9X, RYK, CSNK1A1, SAMD4B, HIPK1, MYO9A, GATAD2A, ABI1, UBAP1, RAB3GAP1, OTUD4, ZNF451, SCARB1, UBE2D3, ARID4B, YY1, RBMX2, KHSRP, CHP2, ZNF569, AREL1, MMGT1, DYRK1A, SSU72, CLEC4D, ZFP42, SRSF3, PHIP, GLYR1, KMT2D, KMT2A, YTHDC1, DIABLO, KMT2C, STK3, ORC2, SKIL, NADK, ZBTB18, NEK9, AKIRIN2, CBX5, CPSF1, PHF12, STYX, HSPA6, FAM217A, ARPC5, AP4B1, CDK8, GNL3L, CDK6, P4HA1, GNAQ, CNOT2, SP3, CDK2, MDM2, NF1, ZNF655, LHX4, OGT, CREBZF, THRA, CELF1, ATP2A3, PTPN21, CTDSPL2, AFF4, SPRED2, SIN3A, PPP6R3, CASP2, TNPO3, METTL17, ACTR2, RBM14, RBM15, KSR1, DST, MMP3, HTR3C, ARNT, WIPI2, PATL1, SENP1, TAPBP, LATS1, UBE2R2, ASPH, IRF6, PPP1R12B, RBM22, SMARCD2, UHRF2, ATF6B, FOXK2, AMBRA1, PARL, ERICH5, GNA13, CDC42, MAPK9, PARD6B, POLR2A, TNKS2, CCL5, TDG, TP53INP1, MAPK1, CLASP2, RBM39, WTAP, TAF13, EYA3, USP22, FBXL17, PDE4DIP, CFLAR, MUC5B, MFSD14C, SCD, FOLR1 | 9.68E-10 | 1.265761719 |
| GO:0046872~metal ion binding | 19.5 | KMT2D, ZNF451, LMAN2L, UHRF2, KMT2C, GNAI3, ATP2A3, ZNF518A, IKZF2, YY1, GNA13, FGD4, FGD6, TNKS2, POLR2A, AKT2, ANKFY1, PMPCB, ZNF569, NADK, ZBTB18, MAP3K2, ZNF561, NEK9, MBNL1, CNOT6L, ZNF460, ZRANB2, KSR1, ESCO1, SCAF11, EYA3, ZNF160, PHF12, MYO9A, MEX3C, RC3H2, TTLL5, CLEC4D, GNAQ, SP3, ZFP42, CDK2, MDM2, LHX4, ZNF780A, ZNF655, RBM22 | 0.004712 | 1.480174474 |
| GO:0005524~ATP binding | 14.2 | PANK2, SRCAP, UBE2D3, ATP2A3, STK3, MAPK9, AKT2, TDG, AKT3, MAPK1, NADK, EPHB3, MAP3K2, NEK9, ACTR2, DYNC1H1, KSR1, CSNK1A1, RYK, HSPA6, ITPK1, DYRK1A, EIF2AK2, HIPK1, MYO9A, HIPK3, UHMK1, GAK, LATS1, CDK8, TTLL5, CDK6, UBE2R2, CDK2, TTLL12 | 5.73E-04 | 1.846713892 |
| GO:0003723~RNA binding | 12.6 | OTUD4, DAZAP1, RPL10, CELF1, YTHDC1, KMT2C, YY1, POLR2A, SIN3A, RBMX2, KHSRP, SRSF11, RBM39, DYNC1H1, RBM14, MBNL1, RBM15, ZRANB2, SCAF11, EIF2AK2, SAMD4B, PUM1, MEX3C, RC3H2, PATL1, UHMK1, GNL3L, HNRNPK, EIF5, SRSF3, RBM22 | 0.003468 | 1.731214948 |
| GO:0003677~DNA binding | 10.5 | CREBZF, KMT2D, KMT2A, SRCAP, UHRF2, EPAS1, KMT2C, ARID4B, YY1, POLR2A, SIN3A, TDG, KHSRP, MAPK1, DEDD, ZBTB18, TAF13, TOP3A, ARNT, HIPK1, RC3H2, PLSCR1, HNRNPK, IRF6, LHX4, GLYR1 | 0.012637 | 1.667435432 |
| GO:0004712~protein serine/threonine/tyrosine kinase activity | 7.7 | MAP3K2, NEK9, KSR1, CSNK1A1, DYRK1A, EIF2AK2, HIPK1, HIPK3, STK3, UHMK1, GAK, LATS1, CDK8, MAPK9, CDK6, AKT2, AKT3, CDK2, EPHB3 | 6.35E-06 | 3.590355381 |
| GO:0004672~protein kinase activity | 6.9 | MAP3K2, NEK9, KSR1, CSNK1A1, RYK, DYRK1A, EIF2AK2, HIPK1, HIPK3, STK3, GAK, CDK8, MAPK9, CCL5, AKT2, AKT3, CDK2 | 1.71E-05 | 3.673781892 |
| GO:0004674~protein serine/threonine kinase activity | 6.9 | MAP3K2, NEK9, KSR1, CSNK1A1, DYRK1A, EIF2AK2, HIPK1, HIPK3, STK3, UHMK1, GAK, LATS1, MAPK9, AKT2, AKT3, CDK2, MAPK1 | 2.85E-05 | 3.523831611 |
| GO:0019899~enzyme binding | 4.8 | RB1, AKIRIN2, CYP2A6, PLSCR1, CPSF1, TNKS2, USP22, HSPA6, MDM2, CASP2, PDE4DIP, UHMK1 | 0.007625 | 2.552526008 |
| GO:0019904~protein domain specific binding | 4.0 | NCOA2, HNRNPK, THRA, TDG, CDK2, MDM2, GNAI3, CASP2, AIDA, SKIL | 0.005405 | 3.077856487 |
| GO:0003729~mRNA binding | 3.6 | RBM14, HNRNPK, RBM15, CELF1, YTHDC1, KHSRP, SAMD4B, PUM1, RC3H2 | 0.007983 | 3.152149575 |
| GO:0008134~transcription factor binding | 3.6 | RB1, MAPK9, UHRF2, THRA, EPAS1, TDG, NAB1, ARNT, MAPK1 | 0.040191 | 2.328976756 |
| GO:0043565~sequence-specific DNA binding | 3.6 | ZBTB18, THRA, EPAS1, ARNT, FOXK2, PRDM2, IRF6, LHX4, GATAD2A | 0.050133 | 2.222792405 |
| GO:0000287~magnesium ion binding | 3.2 | LATS1, PLSCR1, TDG, ITPK1, CDK2, FOXK2, PCK1, STK3 | 0.023049 | 2.838616987 |
| GO:0000976~transcription regulatory region sequence-specific DNA binding | 3.2 | CREBZF, YY1, KMT2D, THRA, ATF6B, FOXK2, ARID4B, IRF6 | 0.026542 | 2.754420721 |
| GO: CELLULAR COMPONENTS | | | | |
| GO:0005634~nucleus | 52.4 | RB1, OTUD4, ZNF451, PANK2, TRRAP, NAB1, UBE2D3, ARID4B, PRDM2, IKZF2, YY1, RBMX2, KHSRP, AKT2, AKT3, CHP2, ZNF569, EPC1, CCNL1, BTRC, ZNF561, MBNL1, ESCO1, ZNF160, DYRK1A, UHMK1, PHIP, TTLL12, KMT2D, GGNBP2, SRCAP, KMT2A, YTHDC1, EPAS1, KMT2C, STK3, SBNO1, BLOC1S2, ORC2, SKIL, SNX6, ZBTB18, AKIRIN2, NEK9, CBX5, CPSF1, PHF12, STYX, HSPA6, EIF2AK2, ARPC5, FANCE, MEX3C, CDK8, HNRNPK, CDK6, CNOT2, SP3, CDK2, RANBP10, MDM2, NF1, LHX4, ZNF655, OGT, CREBZF, SETD5, THRA, CELF1, SIN3A, RSRC1, PPP6R3, CASP2, NCOA2, ACTR2, RBM14, PCYT1A, RBM15, CNOT6L, DST, TOP3A, ARNT, SENP1, LATS1, PLSCR1, IRF6, ZNF780A, RBM22, PAFAH1B1, SMARCD2, ATF6B, CARNMT1, UHRF2, RPL10, FOXK2, ZNF518A, DLST, AMBRA1, PARL, GNA13, MAPK9, PARD6B, POLR2A, TNKS2, TDG, TP53INP1, MAPK1, SRSF11, MLXIP, RBM39, ZNF460, WTAP, USP9X, RYK, CSNK1A1, TAF13, EYA3, FBXL17, SAMD4B, PDE4DIP, HIPK1, OXR1, HIPK3, GATAD2A, TTLL5, PITHD1, ABI1, POLR3E, FOLR1 | 5.60E-17 | 1.9008296 |
| GO:0005829~cytosol | 39.4 | RB1, OTUD4, PANK2, DENND5B, UBE2D3, ARID4B, GLE1, KHSRP, AKT2, ANKFY1, VPS35, BTRC, EPHB3, MBNL1, AREL1, SSU72, AP1AR, NHLRC2, GLYR1, KMT2A, DIABLO, EPAS1, SLAIN2, STK3, PSMF1, PCK1, NADK, SNX6, NEK9, STYX, HSPA6, EIF2AK2, ARPC5, PUM1, AP4B1, GNL3L, EIF5, CDK6, CNOT2, CDK2, RANBP10, MDM2, NF1, OGT, DAZAP1, THRA, SPRED2, PPP6R3, CASP2, ACTR2, PCYT1A, CNOT6L, KSR1, DST, ITPK1, WIPI2, PATL1, GAK, LATS1, PLSCR1, UBE2R2, IRF6, EXOC5, PPP1R12B, ARHGEF7, CD44, PAFAH1B1, CARNMT1, RPL10, DLST, AMBRA1, GNA13, CDC42, FGD4, MAPK9, PARD6B, HECTD2, TNKS2, TP53INP1, MAPK1, DUOX2, CLASP2, MAP3K2, DYNC1H1, USP9X, CSNK1A1, FBXL17, SAMD4B, CFLAR, HIPK1, MYO9A, HIPK3, TTLL5, ABI1, POLR3E, UBAP1, RAB3GAP1 | 1.19E-06 | 1.5467397 |
| GO:0005654~nucleoplasm | 36.9 | RB1, CREBZF, SETD5, DAZAP1, THRA, CELF1, TRRAP, NAB1, UBE2D3, ARID4B, PRDM2, CTDSPL2, AFF4, YY1, SIN3A, AKT2, KHSRP, PPP6R3, EPC1, BTRC, METTL17, NCOA2, MBNL1, RBM14, RBM15, DST, ESCO1, SCAF11, TOP3A, SSU72, DYRK1A, ARNT, WIPI2, SENP1, UHMK1, PLSCR1, SRSF3, IRF6, LETMD1, PPP1R12B, ERGIC1, RBM22, GLYR1, KMT2D, SMARCD2, KMT2A, SRCAP, UHRF2, YTHDC1, EPAS1, KMT2C, FOXK2, DLST, MAPK9, POLR2A, TDG, ORC2, TP53INP1, MAPK1, PSMF1, TAF9B, SKIL, SRSF11, ZBTB18, MAP3K2, AKIRIN2, RBM39, CBX5, ZRANB2, CPSF1, WTAP, TAF13, USP22, PHF12, EYA3, STYX, FBXL17, FANCE, HIPK1, PUM1, GATAD2A, CDK8, GNL3L, HNRNPK, CDK6, CNOT2, SP3, POLR3E, CDK2, MDM2, OGT | 2.38E-12 | 2.038886 |
| GO:0005737~cytoplasm | 36.9 | SCARB1, CELF1, PTPN21, GLE1, YY1, RSRC1, KHSRP, AKT3, PPP6R3, CHP2, CASP2, BTRC, TNPO3, NCOA2, ACTR2, MBNL1, RBM14, AREL1, CNOT6L, KSR1, DST, ITPK1, DYRK1A, ARNT, SENP1, GAK, CYP2A6, PLSCR1, BIN3, ZFP42, SRSF3, TTLL12, EXOC5, IRF6, AIDA, PPP1R12B, RBM22, PAFAH1B1, CDC42SE1, GGNBP2, EPAS1, GNAI3, AMBRA1, STK3, GNA13, CDC42, FGD4, MAPK9, FGD6, POLR2A, TNKS2, MAPK1, PSMF1, PCK1, SKIL, DEDD, SNX6, CLASP2, MAP3K2, DYNC1H1, WTAP, CSNK1A1, USP9X, RYK, EYA3, STYX, HSPA6, FBXL17, SAMD4B, PDE4DIP, EIF2AK2, CFLAR, ARPC5, HIPK1, PUM1, MYO9A, MEX3C, HIPK3, EIF5, HNRNPK, CDK6, PITHD1, GNAQ, CNOT2, CDK2, RANBP10, NF1, MDM2, UBAP1, ZNF655, RAB3GAP1 | 3.66E-05 | 1.4627039 |
| GO:0016020~membrane | 19.1 | DPP10, LMAN2L, RPL10, DENND5B, CELF1, GNAI3, DLST, PTPRK, SLC7A1, CDC42, GLE1, GNA13, SPRED2, KHSRP, ORC2, AKT3, CASP2, ANKFY1, PSMF1, MUC4, CLASP2, ACTR2, DYNC1H1, KSR1, DST, MMGT1, CSNK1A1, USP9X, RYK, KREMEN1, EIF2AK2, WIPI2, RC3H2, TAPBP, GAK, PLSCR1, GNL3L, HNRNPK, P4HA1, SCD, GNAQ, CNOT2, NF1, CALU, AIDA, FOLR1, ERGIC1 | 9.73E-04 | 1.6129168 |
| GO:0032991~macromolecular complex | 8.9 | NCOA2, RBM39, DAZAP1, SMARCD2, CBX5, KSR1, RPL10, SRCAP, SLC51A, WIPI2, SLC7A1, STK3, CDC42, CDK8, PARD6B, AKT2, MDM2, MAPK1, ARHGEF7, SKIL, OGT, RAB3GAP1 | 5.71E-05 | 2.7395386 |
| GO:0005794~Golgi apparatus | 8.9 | SLC30A6, ATF6B, LMAN2L, SRCAP, MMGT1, TRRAP, GNAI3, PDE4DIP, AP1AR, PRDM2, FGFRL1, UHMK1, GAK, FGD4, PLSCR1, FGD6, GNAQ, CALU, MAPK1, CD44, RAB3GAP1, CLASP2 | 0.0144697 | 1.7488567 |
| GO:0000785~chromatin | 7.3 | RB1, CREBZF, MLXIP, NCOA2, AKIRIN2, SMARCD2, ATF6B, ESCO1, THRA, EPAS1, ARNT, FOXK2, FANCE, HNRNPK, SIN3A, SP3, IRF6, LHX4 | 0.0856719 | 1.5147236 |
| GO:0005813~centrosome | 6.9 | NEK9, DYNC1H1, CSNK1A1, EYA3, GNAI3, PDE4DIP, SLAIN2, HIPK1, CDC42, GLE1, TTLL5, CDK6, BLOC1S2, ORC2, CDK2, ARHGEF7, PAFAH1B1 | 5.64E-04 | 2.7178219 |
| GO:0016607~nuclear speck | 6.5 | ZBTB18, RBM39, RBM14, RBM15, WTAP, YTHDC1, CSNK1A1, EPAS1, DYRK1A, HIPK1, PATL1, GATAD2A, RSRC1, SRSF3, CCNL1, SRSF11 | 1.09E-04 | 3.306319 |
| GO:0043231~intracellular membrane-bounded organelle | 6.5 | SCARB1, FOXK2, SNX13, PTPRK, AMBRA1, MUC5B, RC3H2, GAK, CYP2A6, P4HA1, AKT2, UBAP1, ANKFY1, EPC1, ERGIC1, TNPO3 | 0.0997637 | 1.5319278 |
| GO:0005925~focal adhesion | 5.2 | ACTR2, DST, AMBRA1, ARPC5, SENP1, CDC42, GNA13, GAK, HNRNPK, MAPK1, ARHGEF7, CD44, CLASP2 | 0.0038391 | 2.654555 |
| GO:0016605~PML body | 4.4 | RB1, ZNF451, CBX5, TDG, TP53INP1, TOP3A, SP3, HIPK1, SKIL, HIPK3, PATL1 | 4.23E-07 | 8.9422674 |
| GO:0005667~transcription factor complex | 4.0 | RB1, YY1, NCOA2, RBM14, EPAS1, EYA3, ZFP42, CDK2, ARNT, SKIL | 0.0013843 | 3.7794272 |
| GO:0031965~nuclear membrane | 3.2 | GLE1, RBM15, WTAP, GNAQ, ATP2A3, EPC1, SENP1, PAFAH1B1 | 0.0210073 | 2.8965022 |
| KEGG PATHWAYS | | | | |
| hsa05200:Pathways in cancer | 6.0 | RB1, EPAS1, GNAI3, ARNT, CALML4, CDC42, GNA13, MAPK9, CDK6, AKT2, GNAQ, AKT3, CDK2, MDM2, MAPK1 | 0.016409 | 1.992630803 |
| hsa05163:Human cytomegalovirus infection | 5.2 | RB1, ATF6B, GNAI3, CALML4, TAPBP, GNA13, CDK6, CCL5, AKT2, GNAQ, AKT3, MDM2, MAPK1 | 7.03E-05 | 4.075594203 |
| hsa05131:Shigellosis | 5.2 | ACTR2, UBE2D3, ARPC5, WIPI2, CDC42, MAPK9, CCL5, AKT2, AKT3, MDM2, MAPK1, BTRC, CD44 | 1.71E-04 | 3.712585812 |
| hsa05165:Human papillomavirus infection | 4.8 | RB1, CDC42, PARD6B, CDK6, CSNK1A1, AKT2, AKT3, CDK2, MDM2, ATP6V0A2, MAPK1, EIF2AK2 | 0.006669 | 2.557309865 |
| hsa04934:Cushing syndrome | 4.4 | RB1, KMT2D, SCARB1, CDK6, KMT2A, ATF6B, GNAQ, CDK2, GNAI3, ARNT, MAPK1 | 6.01E-05 | 5.006002805 |
| hsa04218:Cellular senescence | 4.4 | RB1, CDK6, AKT2, AKT3, CDK2, MDM2, MAPK1, CALML4, HIPK1, BTRC, HIPK3 | 6.35E-05 | 4.973913043 |
| hsa05169:Epstein-Barr virus infection | 4.4 | RB1, MAPK9, CDK6, SIN3A, AKT2, AKT3, CDK2, MDM2, EIF2AK2, CD44, TAPBP | 5.25E-04 | 3.841239776 |
| hsa05010:Alzheimer disease | 4.4 | MAPK9, CSNK1A1, AKT2, GNAQ, AKT3, ATP2A3, MAPK1, EIF2AK2, WIPI2, CALML4, AMBRA1 | 0.043211 | 2.020652174 |
| hsa05417:Lipid and atherosclerosis | 4.0 | CDC42, MAPK9, CYP2A6, CCL5, AKT2, AKT3, MMP3, HSPA6, MAPK1, CALML4 | 0.003185 | 3.280889788 |
| hsa05132:Salmonella infection | 4.0 | CDC42, ACTR2, MAPK9, DYNC1H1, AKT2, AKT3, ABI1, MAPK1, EXOC5, ARPC5 | 0.008234 | 2.832896805 |
| hsa05135:Yersinia infection | 3.6 | CDC42, ACTR2, MAPK9, AKT2, GNAQ, AKT3, MAPK1, ARPC5, ARHGEF7 | 6.60E-04 | 4.633957474 |
| hsa04915:Estrogen signaling pathway | 3.6 | NCOA2, ATF6B, AKT2, GNAQ, AKT3, HSPA6, GNAI3, MAPK1, CALML4 | 6.92E-04 | 4.600378072 |
| hsa05160:Hepatitis C | 3.6 | RB1, SCARB1, CDK6, AKT2, AKT3, CDK2, MAPK1, EIF2AK2, CFLAR | 0.001599 | 4.04364442 |
| hsa04022:cGMP-PKG signaling pathway | 3.6 | GNA13, ATF6B, AKT2, GNAQ, AKT3, GNAI3, ATP2A3, MAPK1, CALML4 | 0.00236 | 3.801510023 |
| hsa05203:Viral carcinogenesis | 3.6 | RB1, CDC42, HNRNPK, CDK6, ATF6B, CDK2, MDM2, MAPK1, EIF2AK2 | 0.007876 | 3.11202046 |

Table.S4. Details of alteration types and alteration frequencies in the three CRC subtypes resulted from the different expression of the 24 UC-critical genes.

| Alteration Types | Frequency |
| --- | --- |
| Colon Adenocarcinoma | |
| Mutation | 8.2% |
| Amplification | 1.64% |
| Higher mRNA expression | 18.03% |
| Lower mRNA expression | 13.11% |
| Multiple Alterations | 44.26% |
| Mucinous adenocarcinoma of colon and rectum | |
| Mutation | 8.2% |
| Amplification | 1.64% |
| Higher mRNA expression | 18.03% |
| Lower mRNA expression | 13.11% |
| Multiple Alterations | 44.26% |
| Rectal adenocarcinoma | |
| Mutation | 3.87% |
| Structural Variant | 0.65% |
| Amplification | 5.81% |
| Deep Deletion | 0.65% |
| Higher mRNA expression | 10.97% |
| Lower mRNA expression | 21.94% |
| Higher protein expression | 2.58% |
| Lower protein expression | 1.94% |
| Multiple Alterations | 21.29% |
