# Supplementary material for: Identification of genes critical for inducing ulcerative colitis and exploring their tumorigenic potential in human colorectal carcinoma
Source: PLoS One. 2023 Aug 3;18(8):e0289064. doi: 10.1371/journal.pone.0289064 (PMC10399749; doi:10.1371/journal.pone.0289064)
Supplement: Attachment — Submitted filename: renamed_ec270.pdf [file pone.0289064.s004.pdf]

PONE-D-22-35370R1 – Re-revision- review-

=====xxxx===

1. Title: Identification of genes critical for inducing ulcerative colitis ~~in human~~ and exploring their ~~relative~~ ~~influence~~ tumorigenic potential ~~on the tumorigenesis~~ ~~of~~ in human colorectal carcinoma.
2. Figures (graphs/pictures) are still not in the best resolution. Labels and axis details are not clearly visible. I do not know if a separate PDF /or image file is submitted to the journal- where it is visible. Please check.
3. Line-66-67 – p-value adjustments – what do you mean by p-value adjustments?
   Authors' Responses: Gracious thanks to the learned reviewer for the valuable comment. The p-value adjustment signifies the p-value obtained after adjustment of multiple testing. It mainly demonstrates the primary statistics for interpretation of the result and smaller the value the most reliable is the result.

   Reviewer-response- It is well known and understood, what P-value signifies and how it is derived. However from your explanation or mention of "p-value adjustment" as you described as "p-value obtained after "adjustment of multiple testing" - generates or leads reader to confusion – by the word –"adjustment" – This needs to be explained and defined. How this adjustment is achieved in statistical terms? What are the statistical parameters of adjustment? Why was such an adjustment needed? Which "multiple testing" is eligible to adjustment? Perhaps, explain in methodology section -what is meant here by – multiple testing ?What is the threshold of adjustment from the actual value /number of observation?

4. The criteria of selection of UC-critical genes:
5. Line 287- "generating" – developing - // Fuel -> inducing

6. 334-337 -

   Mutation in the alterations of UC-genes critical genes for UC/UC-CRC alter the activation of Wnt, RTK-RAS, TGF-β, and their impact onTP53 signaling and influence the survivability of the affected subjects :

   The results only demonstrate the associated mutations in Wnt, RTK-RAS and TGF-B in this condition, the relative alteration in the activation can only be speculated, therefore it can be only discussed in the discussion and can't be mentioned in the result section title. However, it is appropriate to mention the mutations and the co-relation of mutation with the survival in the title. Please revise the statements accordingly.
   To show the alteration of activity in the function of Wnt, RTK-RAS, TGF-β needs to be demonstrated by functional cell based assays, therefore present accordingly.

7. #386 -389 Transcriptional and translational expression, as well as epigenetic regulation, and proteomics of WFDC2, TTLL12, THRA, and EPHB3 genes preferentially promote the transformation of UC to CRC
   It could be better represented as following:
   => "Expression of WFDC2, TTLL12, HRA and EPHB3 genes and their epigenetic regulation preferentially promote the transformation of UC to CRC'' (only if it is reflected in the presented data)

8. #499 ->"betweenness" – explain the significance in biological term – rather than in Software terminology of a particular package.

9. #524-525- "upregulation and downregulation of 250 genes" -> This statement could be reframed – stating how many upregulated and how many downregulated, respectively. Otherwise just state "differential expression of 250 genes was observed''

10. Immune cell infiltration : Infiltration of immune cells at the site of UC is expected, UC is the result of over-active /unguided neutrophile/macrophase activity. However, the findings of higher expression of the following genes , "*PCK1, WFDC2, MUC4, TTLL12, THRA, EPHB3, DPP10*, and *HSAP6'' ; do they show a gradient of expression in UC samples to increased /elevated expression in CRC samples ?*

11. #587 – Eph : Please do mention complete name – when emphasizing on a particular protein .
