# Supplementary material for: Identification of genes critical for inducing ulcerative colitis and exploring their tumorigenic potential in human colorectal carcinoma
Source: PLoS One. 2023 Aug 3;18(8):e0289064. doi: 10.1371/journal.pone.0289064 (PMC10399749; doi:10.1371/journal.pone.0289064)
Supplement: Attachment — Submitted filename: PONE-D-22-35370.docx [file pone.0289064.s002.docx]

PONE-D-22-35370

Title:

An oncogenomic trajectory study on the identification of ulcerative colitis-critical genes
and their relative influence over the tumorigenesis of human colorectal carcinoma

OR-

Identification of genes critical for ulcerative colitis as inducer of tumorigenesis of colorectal carcinoma.

Or

Bioinformatics mining of ulcerative-colitis gene expression profiles to decipher the inducer-gene-signature of colorectal carcinoma.

Or you could try something else - if you really want to keep “oncogenomic trajectory” – however then the result section has to be explained in that fashion – which I find might be difficult –considering- in the interest of time.

Language suggestion:

There is a scope for language improvement in the Abstract as well as title of the study, methods and results section: here are some suggestion.

Lin 16-18

Line 20 – recognized – can be replaced with more appropriate terminology used in medical science

Line 42- the (not necessary) further - > @Transformation itself define the next change – therefore further is not necessary.

The following terms could be either defined first in the text or use the standard medical terms used in stating diagnostics stages of UC.

Line 55 – “extensive UC patients” - what do you mean by “extensive UC patients”? Similarly, “extensive colitis patients/samples” !!!

Line 58 – “limited UC” ? And “intensity of UC” - > one could state the degree of UC – or use any grading pattern/term used in UC diagnosis description.

Line-66-67 – p-value adjustments – what do you mean by p-value adjustments?

How do you define UC-critical genes? What do you mean by “critical£ here? Do you want to say differentially regulated / or upregulated /downregulated genes – i.e. an expression profile signatory of a stage in UC marking the transformation into colorectal carcinoma? Once you define it use that term in the rest of the text.

Line -81- “Screening of UC-Critical Genes” - ?

Line -89 – “across c-Bioportal” - using c-Bioportal.

Line 103 – “taking a clue” – this clue could be explained technically – e.g. highly expressed genes / or differentially regulated genes or which observation made it as a clue – that could be explained in brief here.

Line 115 – KM plot- mentioned for first time – therefore, use long form followed by bracket (KM)

Line 117 – immune cell infiltration analysis

Line- 132- characteristics of samples selected

Line 500-501 – “as the potential ~~oncogenic~~ mediators of the oncogenic transformation of CRC from UC” // or// “ as the potential oncogenic mediators of transformation of CRC from UC”.

Methods: Please revise for language- refer papers in these genera from previous PLOS one publications in 2022.

Disease occurrence:

1) It would add more information if authors could also add data from their local geographies?

2) Add stats from VizHub – global disease burden study – you could use the figures generated form database.

Is there any data available on the diet of the patients?

Is there a possibility to trace from the database that you used?
