# Supplementary material for: Identification of genes critical for inducing ulcerative colitis and exploring their tumorigenic potential in human colorectal carcinoma
Source: PLoS One. 2023 Aug 3;18(8):e0289064. doi: 10.1371/journal.pone.0289064 (PMC10399749; doi:10.1371/journal.pone.0289064)
Supplement: Attachment — Submitted filename: Reviewers comment.docx [file pone.0289064.s005.docx]

**PONE-D-22-35370 R1**

**"Identification of genes critical for inducing ulcerative colitis in human and exploring their relative influence on the tumorigenesis of colorectal carcinoma"**

Reviewer #1:

The study is good, and the manuscript is taking shape, however some issues needs to be addressed as follows so as to reach audience lucidly, please find my comments attached herewith -in a separate file.
Other than that a major revision is needed to address the resolution of figures. If a separate better quality image file is submitted to the journal, which is not attached with manuscript for review, then choose the correct file. Thank you.

**Response to the reviewer’s comment:**

Grateful thanks to the learned reviewer for appreciating our revised work and for the valuable feedback. The figures provided are of high resolution of 600dpi and are attached separately with the revised manuscript.

1. Title: Identification of genes critical for inducing ulcerative colitis ~~in human~~ and exploring their ~~relative influence~~ tumorigenic potential ~~on the tumorigenesis of~~ in human colorectal carcinoma.

**Authors’ response:**

Sincere thanks to the learned reviewer for revising the title. We have incorporated the changes in the revised manuscript.

2. Figures (graphs/pictures) are still not in the best resolution. Labels and axis details are not clearly visible. I do not know if a separate PDF /or image file is submitted to the journal- where it is visible. Please check.

**Authors’ response:**

Sincere thanks to the learned reviewer for the valuable advice. We have incorporated the high-resolution clear figures separately with the revised manuscript.

3. Line-66-67 – p-value adjustments – what do you mean by p-value adjustments? Authors’ Responses: Gracious thanks to the learned reviewer for the valuable comment. The p-value adjustment signifies the p-value obtained after adjustment of multiple testing. It mainly demonstrates the primary statistics for interpretation of the result and smaller the value the most reliable is the result.

Reviewer-response- It is well known and understood, what P-value signifies and how it is derived. However from your explanation or mention of “p-value adjustment” as you described as “p-value obtained after “adjustment of multiple testing” - generates or leads reader to confusion – by the word –“adjustment” – This needs to be explained and defined. How this adjustment is achieved in statistical terms? What are the statistical parameters of adjustment? Why was such an adjustment needed? Which “multiple testing” is eligible to adjustment? Perhaps, explain in methodology section -what is meant here by – multiple testing ?What is the threshold of adjustment from the actual value /number of observation?

**Authors’ response:**

Grateful thanks to the learned reviewer for the valuable feedback. We have modified the manuscript by changing the word “p-value adjustment”. The explanation regarding the p-value adjustment is that it is obtained after adjustment of multiple testing. It is performed for reducing the occurrence of false positive results. The statistical method used here is Benjamini & Hochberg false discovery rate method as it provides a good balance between discovery of statistically significant genes and limitation of false positives results. The threshold value for the p-value used here is >0.05. We have incorporated all the necessary changes in the revised manuscript.

4. The criteria of selection of UC-critical genes:

**Authors’ response:**

Sincere thanks to the learned reviewer for the comment and the correction has been made within the revised manuscript.

5. Line 287- “generating” – developing - // Fuel -> inducing

**Authors’ response:**

Sincere thanks to the learned reviewer for the valuable correction. We have incorporated the changes in the re-revised manuscript.

6. 334-337 - Mutation in the alterations of UC-genes critical genes for UC/UC-CRC alter the activation of Wnt, RTK-RAS, TGF-β, and their impact on TP53 signaling and influence the survivability of the affected subjects : The results only demonstrate the associated mutations in Wnt, RTK-RAS and TGF-B in this condition, the relative alteration in the activation can only be speculated, therefore it can be only discussed in the discussion and can’t be mentioned in the result section title. However, it is appropriate to mention the mutations and the co-relation of mutation with the survival in the title. Please revise the statements accordingly. To show the alteration of activity in the function of Wnt, RTK-RAS, TGF-β needs to be demonstrated by functional cell based assays, therefore present accordingly.

**Authors’ response:**

Sincere thanks to the learned reviewer for valuable suggestion. We have modified the re-revised manuscript accordingly.

7. #386 -389 Transcriptional and translational expression, as well as epigenetic regulation, and proteomics of WFDC2, TTLL12, THRA, and EPHB3 genes preferentially promote the transformation of UC to CRC It could be better represented as following: => “Expression of WFDC2, TTLL12, HRA and EPHB3 genes and their epigenetic regulation preferentially promote the transformation of UC to CRC’’ (only if it is reflected in the presented data)

**Authors’ response:**

We express our grateful thanks to the learned reviewer for the valuable suggestion. We have incorporated the changes in the re-revised manuscript.

8. #499 ->”betweenness” – explain the significance in biological term – rather than in Software terminology of a particular package.

**Authors’ response:**

Gracious thanks to the learned reviewer for the valuable suggestion. The term betweenness is a scientific term for understanding how often a node occurs on all shortest paths between two nodes of an interacting network. It measures the number of shortest paths going through the nodes. Nodes that have the higher value of betweenness acts as important bottleneck in any network. We have incorporated the changes in the revised manuscript.

9. #524-525- “upregulation and downregulation of 250 genes” -> This statement could be reframed – stating how many upregulated and how many downregulated, respectively. Otherwise just state “differential expression of 250 genes was observed’’

**Authors’ response:**

We express our grateful thanks to the learned reviewer for the valuable suggestion. We have incorporated the changes in the revised manuscript.

10. Immune cell infiltration : Infiltration of immune cells at the site of UC is expected, UC is the result of over-active /unguided neutrophile/macrophase activity. However, the findings of higher expression of the following genes , “PCK1, WFDC2, MUC4, TTLL12, THRA, EPHB3, DPP10, and HSAP6’’ ; do they show a gradient of expression in UC samples to increased /elevated expression in CRC samples ?

**Authors’ response:**

Grateful thanks to the learned reviewer. Yes, we do agree with the learned reviewer that the induction of UC leads to the infiltration of immune cells within the site of induction of inflammation resulting in the overactivation of neutrophil and macrophage activity. Previous studies have shown that expression of PCK1, WFDC2, MUC4, TTLL12, THRA, EPHB3, DPP10 and HSAP6 are correlated with the infiltration of immune cells across the tumor microenvironment for different types of cancer (Yu et al., 2023, He et al., 2022, Cappello et al., 2019, Das et al., 2016). These genes are also associated with the invasion of immune cells in the damaged tissue for ulcerative colitis. Our study has shown that the higher expression of these genes in respect to colorectal cancer patient shows an increase in infiltration of neutrophils, macrophages and NK cells within and around the tumor microenvironment (Figure 5A).

11. #587 – Eph : Please do mention complete name – when emphasizing on a particular protein.

**Authors’ response:**

We express grateful thanks to the learned reviewer for the valuable suggestion. We have incorporated the full form of EPHB3 Erythropoietin-Producing Hepatoma (EPH) receptor B3 in the revised manuscript.
